# Bush encroachment dynamics and rangeland management implications in southern Ethiopia

**DOI:** 10.1002/ece3.4621

**Published:** 2018-10-26

**Authors:** Chuan Liao, Patrick E. Clark, Stephen D. DeGloria

**Affiliations:** ^1^ School of Sustainability Arizona State University Tempe Arizona; ^2^ Northwest Watershed Research Center USDA Agricultural Research Service Boise Idaho; ^3^ School of Integrative Plant Science, Crop and Soil Sciences Section Cornell University Ithaca New York

**Keywords:** bush encroachment, Ethiopia, phenology, rangeland, state‐and‐transition model

## Abstract

Rangelands in southern Ethiopia have been undergoing a rapid regime shift from herbaceous to woody plant dominance in the past decades, reducing indigenous plant biodiversity, altering ecosystem function, and threatening subsistence pastoralism. Despite significant rangeland management implications, quantification of spatial encroachment extent and transitional pathways that result in encroachment remain largely under‐explored. This paper develops a phenology‐based approach to map rangeland vegetation states in southern Ethiopia, and examines transition pathways among states using the state‐and‐transition model. The results indicate that nearly 80% of landscape was dominated by woody plants in 2013. While stable encroached states have been established in both high and low lands through different transition pathways between 2003 and 2013, we identified spatial locations where bush encroachment occurred rapidly. The multiplicity in the transition pathways indicates opportunities for positive transformation in the entire rangeland system in southern Ethiopia and other semi‐arid regions of Africa.

## 1 INTRODUCTION

Rangelands cover 41% of the terrestrial land surface and provide over $1 trillion in ecosystem services to 36% of the global population (FAO, 2001). However, woody plant proliferation, commonly known as bush encroachment, has been a growing concern for rangeland management globally (Anadón, Sala, Turner, & Bennett, 2014; Brandt, Haynes, Kuemmerle, Waller, & Radeloff, 2013; Gartzia, Alados, & Pérez‐Cabello, 2014). Bush encroachment substantially suppresses the growth of high‐value herbaceous forage species in the understory, reduces indigenous plant biodiversity, and alters rangeland ecosystem functions (Rundel, Dickie, & Richardson, 2014; Scholes & Archer, 1997). In addition, reduced forage can threaten subsistence pastoralism that primarily rely on cattle grazing (Smith, Barrett, & Box, 2000).

In the Borana Zone of southern Ethiopia, bush encroachment has been intensifying over the past decades, which is becoming as a serious threat to both rangeland health and the livelihoods of millions of pastoralists (Angassa, 2014; Coppock, 2016). However, our poor spatial understanding of the extent and dynamics of bush encroachment in Borana continues to limit the effectiveness of rangeland management. Previous spatial evaluations produced only aggregate and seemingly contradictory estimates of encroachment extent within the Borana Zone: about 40% of Borana rangelands were estimated as encroached in the 1980s (Coppock, 1994), over 70% in the late 1990s (Oba, Post, Syvertsen, & Stenseth, 2000), and 52% in the 2000s (Gemedo‐Dalle, Maass, & Isselstein, 2006). Such aggregate estimates revealed little about the complex spatial distribution of encroached rangelands or potential encroachment risk. Without a better spatial understanding of encroachment dynamics throughout the entire zone, favorable opportunities for rangeland management and encroachment mitigation may be missed, and substantial efforts and resources can be applied in areas where mitigation stands little chance of success. Filling this spatial information gap is, consequently, both crucial and long overdue.

The complexity of rangeland vegetation dynamics can be interpreted by the state‐and‐transition model, in which rangeland dynamics are described as a set of discrete “states” of vegetation at a specific site and changes between states that occur as discrete “transitions” (Briske, 2017; Milton & Hoffman, 1994; Westoby, Walker, & Noy‐Meir, 1989). Transitions from one state to another often require a combination of climatic circumstances and management actions (e.g., fire or grazing) to bring them about (Mayer & Khalyani, 2011; Staver, Archibald, & Levin, 2011). Knowledge of the spatial distribution of vegetation states across the landscape is needed to appropriately fit intervention strategies to rangeland management practices where they will be most advantageous (D'Odorico, Okin, & Bestelmeyer, 2011). Yet, spatial knowledge of vegetation states alone is insufficient to guide effective management because efficacy also depends on the underlying mechanisms that bring about these states. Therefore, spatial knowledge of current vegetation states plus understanding of past and future transition pathways is needed to properly prescribe and apply efforts to mitigate undesirable processes such as bush encroachment.

The goal of this study was to provide pastoralists, rangeland managers, and policy makers with a spatial understanding of the past, current, and potential rangeland vegetation states in Borana, Ethiopia, which will then contribute to assessing spatial risk of bush encroachment and applying mitigation actions for rangeland management. Our specific objectives were to: (a) develop a phenology‐based remote sensing approach to classify rangelands according to the state‐and‐transition theory, (b) investigate spatial rangeland states and distribution patterns, and (c) examine transition pathways among different states in order to enhance the prediction of vegetation dynamics. Our findings will aid in predicting directionality (and perhaps magnitude) of future vegetation dynamics and serve to improve the efficacy of bush encroachment mitigation efforts by identifying areas or situations to prioritize in southern Ethiopia and other similar semi‐arid areas of Africa.

## 2 METHODS

### 2.1 Study area

**Figure 1:**
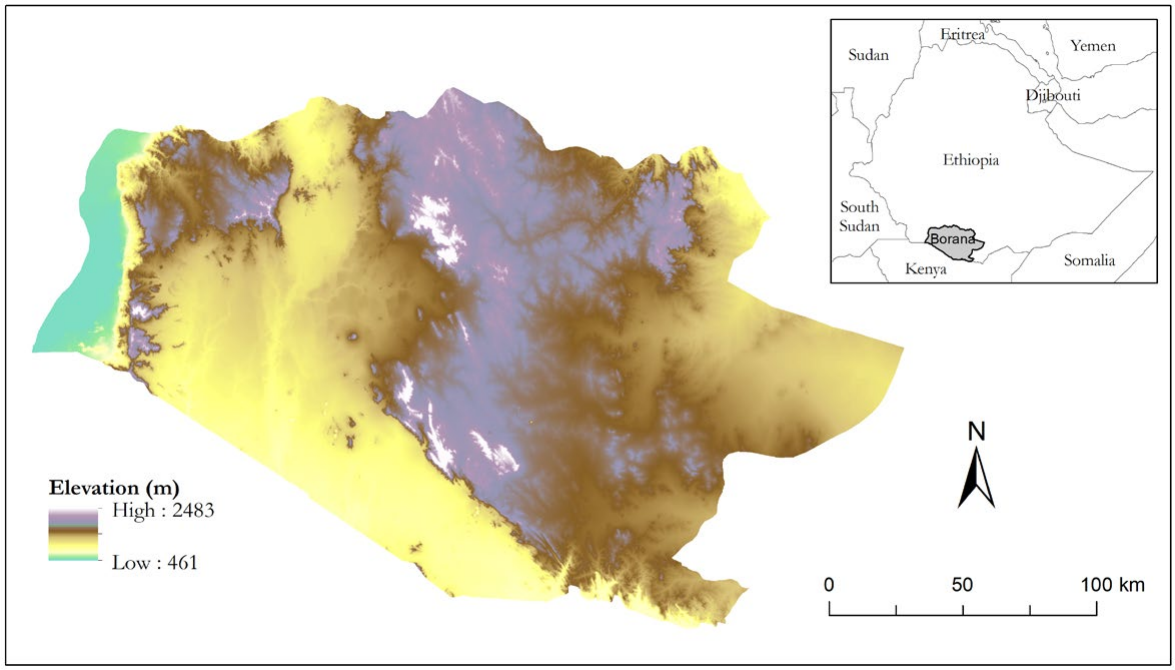
Geographic location and topography of the Borana Zone in southern Ethiopia

This study was conducted in the Borana Zone (~44,000 km^2^) of southern Ethiopia (Figure 1). Elevation here ranges from 500 to 2,500 m above sea level. Terrain varies from flat, dry river and lake beds in the lowlands to steep hillslopes in the highlands. Climate is largely semi‐arid with relatively cool annual temperatures (19–24°C) for this tropical setting. Mean annual rainfall ranges from 300 mm in the lowlands to 1,000 mm in the highlands. Precipitation distribution is bimodal, with 60% in the primary rainy season (April–May) and 30% in the secondary rainy season (October–November). In between the rainy seasons are warm dry season (December–March) and cool dry season (June to September). Generally, annual precipitation is positively correlated with elevation (Coppock, 1994).

### 2.2 Borana rangeland dynamics and hypotheses

Vegetation in Borana is dominated by herbaceous and woody plants in varying composition ratios, with woody plants cover ranging from 5% to 75% (Angassa & Oba, 2008; Coppock, 1994; Tefera, Snyman, & Smit, 2007). Such variability gives rise to a complex and diversely‐vegetated landscape typical of the Horn of Africa (Cossins & Upton, 1987). Like many semi‐arid rangelands, much of the Borana vegetation occurs as transitional phases between stable states of grassland and savanna and between savanna and woodland (Mayer & Khalyani, 2011). Temperature, precipitation, fire, grazing, and soil have all been described as crucial in the origin, maintenance, and shift of vegetation on tropical rangelands (Lehmann, Archibald, Hoffmann, & Bond, 2011; Stevens, Lehmann, Murphy, & Durigan, 2017). While climatic and edaphic factors primarily determine broad‐scale vegetation distribution, complex patches of open and closed canopy rangelands can exist within a single climate zone, suggesting that controls such as fire and herbivory are important at a finer spatial scale (Beard, 1953; D'Odorico et al., 2011; Kraaij & Milton, 2006).

The state‐and‐transition model (Briske, Fuhlendorf, & Smeins, 2003, 2005 ) sheds light on interpreting the evolving nature of Borana rangelands at the local scale. Before the 1970s, fire was a major factor that determined rangeland states and transitions (Angassa & Oba, 2008), which could transform the rangeland into a grass‐dominated system. Subsequently, plant recruitment, herbivory, and low‐intensity fire would contribute to maintaining an open woodland/scrubland state, until the next high intensity fire to bring the entire system back to grassland. After fire ban since the 1970s, however, grazing and plant recruitment gradually transformed grasslands and open woodland into dense thickets. Continued grazing could diminish forage in the understory, thus leaving the scattered woody plants free from competition and accelerating bush encroachment (Liao & Clark, 2018).

Based on published knowledge on the determinants of rangeland vegetation dynamics and the state‐and‐transition model, we propose the following three hypotheses:

H1: Different rangeland vegetation states have distinct topographic niches in terms of elevation and slope.

H2: There are distinct rangeland vegetation states, and due to their unique physiognomy and floristic composition, they demonstrate significantly different phenological characteristics.

H3: The Borana rangelands are an evolving system, as there is a significance difference in vegetation states between 2003 and 2013.

### 2.3 Data collection and rangeland classification scheme

We conducted fieldwork in the Borana Zone 2013 to build the foundation for a rangeland classification scheme. The research team traveled to both highland and lowland areas of the zone to investigate the diversity of rangeland vegetation in terms of physiognomic features, floristic composition, and phenological characteristics. In addition, we conducted vegetation surveys in five study sites that represent a wide range of ecological zones in Borana (see details in supplementary materials). At each site, we surveyed 27 plots and collected data on species composition and vegetation cover. Voucher specimens were collected, and their scientific names identified (Supporting Information Table S1).

A couple of existing classification approaches, which were developed to distinguish and map African vegetation classes, likely have applicability in our study area. The White classification system, which is primarily based on vegetation physiognomy and floristic composition to achieve an objective classification framework, consist of 80 major vegetation types (White, 1983). In East Africa, a combination of the growth‐form type and relative contribution of woody and herbaceous plants can lead to the differentiation of six major vegetation types: (a) bushland, (b) woodland, (c) grassland, (d) bushed grassland, (e) wooded grassland, and (f) dwarf shrub grassland (Pratt, Greenway, & Gwynne, 1966). Based on field vegetation assessment and existing classification approach by Pratt et al. (1966), we developed a classification scheme that includes eight classes representing the vegetation states potentially present in Borana (Table 1).

**Table 1:** Description of vegetation classes in the Borana Zone of southern Ethiopia. Vegetation classes are presented in the order of decreasing woody plant cover or composition

Vegetation class	Code	Description
Closed Canopy Woodland	CCW	A stand of trees with an interlaced canopy, usually with shrubs interspersed, and a tree canopy cover over 50%. CCW is usually distributed in relatively humid areas. Dominant tree species include *Juniperus procera*,* Combretum molle,* and *Terminalia brownie*
Dense Scrubland	DS	An assemblage of woody plants, mostly of shrubby habit, having a shrub canopy cover over 50%. The aerial cover by grasses and herbs is at a low to intermediate level, between 20% and 40%. Dominant woody species include *Acacia drepanolobium*,* A. nilotica,* and *A. hockii*
Bushland	BU	Land dominated by shrubs with scattered trees, with woody plant cover around 50%. The herbaceous plant cover is usually <30%. BU is usually distributed in relatively arid areas, but it has the potential to shift into DS as canopy closes. Dominant woody species include *A. mellifera* and *A. reficiens*
Open Canopy Woodland	OCW	A stand of trees with open canopy, usually with few shrubs interspersed, and a canopy cover <40%. The aerial cover by grasses and herbs is at an intermediate level between 20% and 50%. When the canopy layer thickens, it can shift into DS or CCW. Dominant tree species include *A. tortilis* and* Commiphora africana*
Sparse Scrubland	SS	Land with scattered or grouped shrubs. The shrubs are always conspicuous, but trees are sparse, with a canopy cover between 10% and 30%. Growth of grasses is somewhat suppressed, with an aerial cover between 10% and 20%. This vegetation class represents the state between GR and DS. Major woody species include *Solanum giganteum* and *Hibiscus boranensis*
Cultivated Land	CL	Land being used for crop cultivation. Common crops include *Zea* sp, *Sorghum* sp and *Erogrostis tef*. CL is usually fenced and located in relatively wet places, and sometimes close to seasonal rivers
Grassland	GR	Land dominated by grasses and occasionally other herbs, with an aerial cover over 40%. The widely scattered trees and shrubs can contribute to a canopy cover that rarely exceeds 10%. Major herbaceous species include *Cenchrus ciliaris*,* Chrysopogon auheri*,* Enneapogon persicus*, and *Panicum maximum*
Sparsely Vegetated Land	SV	Land poorly covered by either herbaceous or woody plants, with plant cover rarely exceeds 10%. SV either represents the result of heavy grazing on the newly established grassland, or merely bare ground dominated by rock, sand or lava. The top soil is usually lost due to low basal cover

In order to link remotely‐sensed vegetation indices with contextualized vegetation states, we collected geo‐referenced photos of representative rangeland vegetation classes throughout the entire Borana Zone. The sample we collected was distributed across the Borana Zone covering both low and high lands (Supporting Information Figure S1). The vegetation types recorded by these photographs were classified based on the criteria in Table 1. A total of 1,866 valid samples were collected, and they were interpreted as eight classes including 85 CCW, 278 OCW, 615 DS, 256 SS, 388 BU, 68 GR, 172 CL, and 4 SV.

We referred to both spectral and temporal traits of satellite imageries to map the spatial distribution of existing vegetation states in the Borana Zone. Specifically, we used the Normalized Difference Vegetation Index (NDVI) images with a spatial resolution of 250 m and temporal resolution of 16 days, which has proven to be effective for rangeland classification (Piao, Mohammat, Fang, Cai, & Feng, 2006). Since the training points were collected in the year of 2013, we used the 23 images from that year in our classification. We also obtained the 23 images in 2003 to derive the vegetation states a decade prior and compare those with 2013 conditions (Supporting Information Table S2).

### 2.4 Data analysis

In order to capitalize on phenological differences existing among differing vegetation states for rangeland classification, we conducted supervised classification using the non‐parametric random forest classifier. The classification was performed using the randomForest package (Liaw & Wiener, 2002) in the R software environment (R Development Core Team, 2014). In this study, the 23 NDVI images available throughout the year were considered 23 predictor variables. We randomly selected 75% of ground‐truth data for use in training the classifier, and the remainder were used to assess the accuracy of resultant classification map by constructing a classification error matrix.

We also investigated the phenological features of each vegetation class. Generalized additive model (GAM) was used to investigate the phenological features of vegetation classes. The model can be represented as:

$$\left[\text{NDVI}\;=\;\beta_{0}\;+\;f\left(\text{Date}\right)\;+\;\beta_{1}\;\text{VegeClass}\;+\;\varepsilon ,\right]$$

where *β*_0_ is the intercept; *f*(Date) is non‐linear and is subject to smoothing splines; *β*_1_ is coefficient of vegetation class; *ε* is the error and *ε* ~ *N*(0, s^2^). The analysis was conducted at the pixel level. However, there is a total of 688,100 pixels within Borana Zone, and this huge sample size was beyond the computational power of R software. Therefore, we randomly selected 1% pixels to perform GAM. Since the mean NDVI curve of open canopy woodland is the closest to sample mean, we used it as the benchmark factor in the model.

We used a digital elevation model (DEM) having a spatial resolution of 3‐arc seconds (~90 m) to evaluate the topographic characteristics of each vegetation class. From this DEM raster image, we also estimated slope gradient in percent. Then, we overlaid the topographic raster images with the predicted rangeland map, and extracted the elevation and percent slope gradient for each classified vegetation at the pixel level. We conducted ANOVA to test whether there is any significant difference on the topographic characteristics among the eight vegetation classes.

In order to identify vegetation transition pathways from 2003 to 2013, we conducted a pixel‐level change detection analysis (Tewkesbury, Comber, Tate, Lamb, & Fisher, 2015). We first constructed a vegetation change matrix from the two classified vegetation raster images in these two years, and then summarized the pixel‐level transition from one vegetation class to another. We used the Gmisc package (Gordon, 2016) in the R statistical software to create a vegetation transition plot.

## 3 RESULTS

### 3.1 Spatial vegetation classes and phenological features

**Figure 2:**
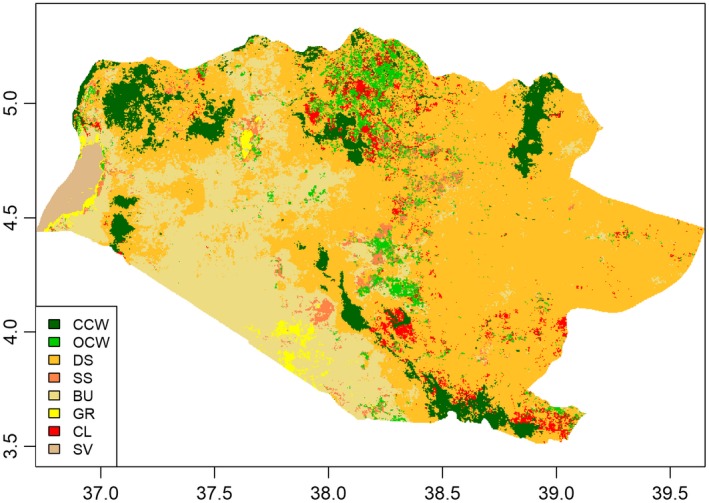
Vegetation classes and their spatial distribution in 2013. Dense scrubland and bushland dominated the landscape, accounting for over 80% of the entire zone

**Figure 3:**
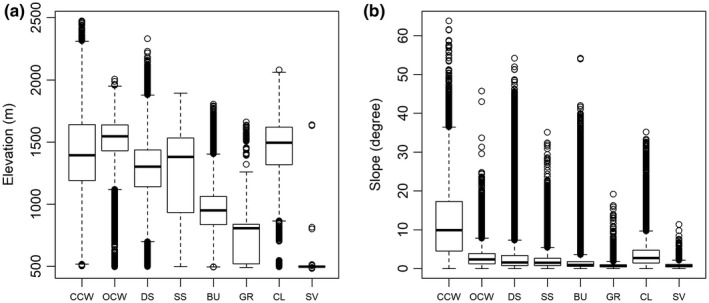
Elevation (a) and slope (b) range of each vegetation class. Results from ANOVA indicate the topographic characteristics of these eight classes are significantly different from each other (*p*‐value < 0.001)

The classification results indicated substantial variation in the spatial distribution patterns of eight vegetation classes across the Borana Zone in 2013 (Figure 2). Validation of classification results revealed an overall classification accuracy at 76.1% (Supporting Information Table S3). These classes are present in differentiated topographic characteristics in terms of elevation (*F*_7, 687,894_ = 58,939, *p*‐value < 0.001) and slope (*F*_7, 687,894_ = 26,224, *p*‐value < 0.001; Figure 3).

In 2013, the two vegetation classes dominated by woody plants contributed to over 80% of total land area. As the most expansive class, dense scrubland covered 57.8% of the zone. This class was mainly distributed in the eastern and northern part of Borana, with an average elevation around 1,350 m. The other type of dominant class, bushland, covered 23.2%. In contrast to dense scrubland, bushland was mostly distributed in the lowland in the southwest with an average elevation at approximately 950 m.

The remaining 20% of the zone was covered by the other six vegetation classes. Closed canopy woodland, representing 7.6% of the total area, primarily occupied the highland area above 1,400 m. Open canopy woodland, which was the savanna‐like class, represented merely 3.8% of landscape. Once the dominant vegetation class on the Borana rangelands (Cossins & Upton, 1987), patches of open canopy woodland were largely restricted to the north and central part of the zone. Cultivated land accounted for 3.0% of landscape. In general, cultivated land tended to appear in the highlands, with a mean elevation around 1,500 m. Sparse scrubland, represented by scattered shrubs and some grass, occupied 2.2% of total area. Sparsely vegetated land, which represented 1.4% of total area, was mostly distributed in the far western corner of Borana. It had the lowest average elevation at 510 m. Grassland was the least common vegetation class. It occupied 1.1% of landscape, and was mainly distributed in the lowland flat area.

The eight vegetation classes exhibit distinct phenological features (Table 2). Values of NDVI varied throughout the year, as shown by the highly significant Date effect. All vegetation classes were significantly different from the reference class (open canopy woodland), although the magnitude of these differences varied. In general, classes with higher woody plant cover than open canopy woodland demonstrated positive coefficient, while those with less woody plants showed negative estimates. The only exception was cultivated land.

**Table 2:** Estimation of Normalized Difference Vegetation Index values for eight vegetation classes using GAM

Variables	Estimate	*SE*	*t* Value
Intercept	0.309***	0.002	147.830
Date	0.003***	0.000	45.797
CCW	0.215***	0.002	90.553
DS	0.094***	0.002	45.978
BU	0.025***	0.002	12.000
SS	−0.015***	0.003	−4.492
CL	0.054***	0.003	19.097
GR	−0.121***	0.004	−30.506
SV	−0.243***	0.004	−68.488

a Indicates significant at 0.001 level.

**Figure 4:**
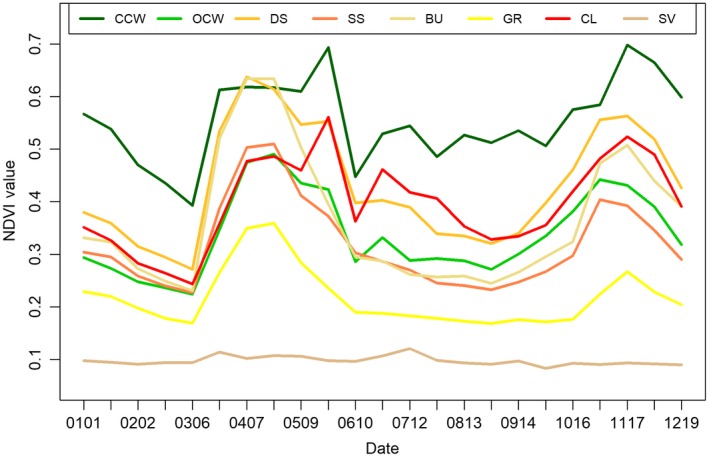
Mean Normalized Difference Vegetation Index curves of eight vegetation classes

Mean NDVI curves of eight vegetation classes illustrated their unique phenological features (Figure 4). Among these eight curves, sparsely vegetated land and grassland did not intersect with any other ones and represented the two classes with consistently low mean NDVI values across time. In contrast to other curves with two peaks, the curve of sparsely vegetated land fluctuated around 0.1 without showing any peaks during the reason seasons in April and November. Grassland demonstrated a bimodal curve as other six classes, but its mean NDVI value was consistently lower than others at any time of year.

At the higher end of mean NDVI values, closed canopy woodland clearly stood out from the other classes. Except for a few April dates in the major rainy season, its NDVI values were consistently higher than all other classes. It is likely that dense tree canopy contributed to the observed high NDVI values throughout the year.

Normalized Difference Vegetation Index curves for the remaining five classes were much less distinct. Dense scrubland was characterized by two high peaks in the two rainy seasons. It maintained a relatively high NDVI value even in the dry seasons. In contrast, bushland, which was also dominated by shrubs, exhibited lower NDVI values during the dry season despite its high greenness in the rainy season. Since the primary bushland species (*A. mellifera* and *A. reficiens*) are deciduous, this state exhibits a distinct reduction in NDVI during the dry seasons due to leaf drop. Sparse scrubland, with highly scattered shrub layer, demonstrated much lower NDVI in contrast to dense scrubland and bushland throughout the year.

Open canopy woodland, the reference class in the GAM, exhibited a similar NDVI curve to that of sparse scrubland. However, because of its intermediate tree layer cover by species such as *A. tortilis*, open canopy woodland exhibited had higher NDVI values in the cool dry season than sparse scrubland and these distinct temporal signals were exploited to distinguish between these classes.

Cultivated land was the only land cover class that was intensively managed by human beings. Sparse tree and shrub cover on these crop fields made it less green in general. However, since it was under human management during the dry season, it exhibited the second highest NDVI values in this time window, which distinguished it from all other vegetation classes.

### 3.2 Vegetation state transitions

**Figure 5:**
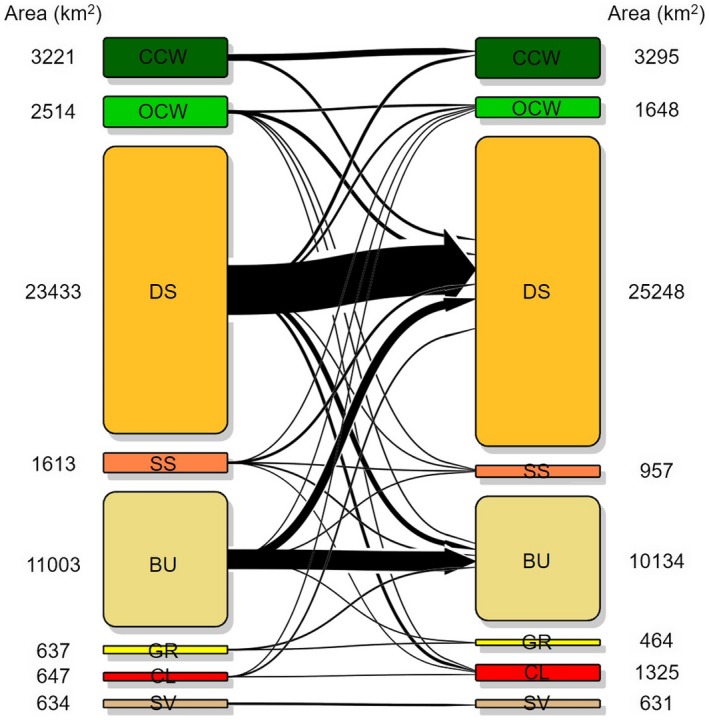
Vegetation transition in the 2003–2013 decade. Transitions with an area <10 km^2^ are not included. See change in each vegetation state in Supporting Information Table S4

In order to detect changes in vegetation classes in the 2003–2013 decade, we generated the rangeland vegetation map in 2003 by using the same algorithm and training data but 2003 imagery (Supporting Information Figure S2). Comparison between 2003 and 2013 vegetation maps indicated that over the decade, while the rank of dominance by areal coverage largely remained the same, substantial expansions and declines occurred between some vegetation classes (Figure 5; Supporting Information Table S4). The chi‐square test of the composition of vegetation classes suggested significant difference between these two years (*χ* ^2^_7, 699,243_ = 11,618, *p*‐value < 0.001).

The vegetation transition diagram revealed the directions of vegetation shifts (Figure 5). Comparison between 2003 and 2013 vegetation maps at the pixel level suggested that 30% of the land in the entire Borana Zone shifted from one class to another. Among the eight vegetation classes, sparsely vegetated land was the most stable. About 99% of pixels classified to sparse vegetation in 2003 remained classified to this class in 2013. Dense scrublands, bushlands, and closed canopy woodlands were also quite stable, where over 70% of pixels originally assigned to these classes did not change class during the 2003–2013 decade.

Extent of the remaining four classes during 2003–2013, however, was more volatile. The general transition pathway in these cases proceeded from a less‐encroached state to a more‐encroached state. For grasslands, only 218 km^2^ out of 637 km^2^ remained in the same class by 2013. The greatest loss of grassland, representing a net decrease of 398 km^2^, was caused by transitions into bushland. For open canopy woodland, 629 km^2^ out of 2,514 km^2^ remained the same. A total of 625 km^2^ of open canopy woodland transitioned into dense scrubland, representing the greatest source of areal decline for this class. Another about 125 km^2^ of open canopy woodland transitioned into bushland and 63 km^2^ were lost to cultivated land by 2013. Sparse scrubland maintained less than a quarter of its area from 2003. Transitions into dense scrubland and bushland represented the greatest loss of extent from this class. Cultivated land was most volatile class where 129 km^2^ out of 1,325 km^2^ in 2013 was from 2003. While this class primarily expanded in extent, due to conversions of dense scrublands (871 km^2^) and open canopy woodlands (185 km^2^) into crop fields during the 2003–2013 decade, 355 km^2^ of cropland transitioned backed to dense scrublands and 124 km^2^ back to open canopy woodlands.

## 4 DISCUSSION

Although earlier research has investigated the diversity of rangeland vegetation and assessed the condition of bush encroachment in southern Ethiopia and other semi‐arid areas of Africa (Angassa & Baars, 2000; Gemedo‐Dalle et al., 2006; Pratt et al., 1966), little empirical work has been conducted to quantify the spatial extent of different vegetation classes and interpret vegetation dynamics. Our analysis of rangeland vegetation states and transition pathways in Borana, while confirming the overall trend of encroachment as highlighted in literature (Coppock, 2016; Gemedo‐Dalle et al., 2006), reveals the spatial distribution of different vegetation classes and the transition pathways that led to the proliferation of woody plants. The findings refuted the perception of Borana rangelands as homogeneously encroached. Instead, the vegetation was highly diverse and varied substantially in its composition of woody and herbaceous plants. In addition, the findings increase our ability to predict which areas are at the nexus of being at a high risk of further encroachment, and shed light on developing spatially explicit strategies for mitigating bush encroachment and improving rangeland management in the arid and semi‐arid environment.

Our integrated approach, which combined field‐based assessment, time‐series NDVI data, and random forest algorithm, represented a significant contribution to mapping bush‐encroached rangelands (Reed, Schwartz, & Xiao, 2009). Despite of minor classification error in the vegetation map of 2013 and potential unknown error in the map of 2003, our approach effectively captured the phenological features to differentiate various vegetation classes. In contrast to the common understanding that rangelands with higher NDVI is associated with better forage (Chantarat, Mude, Barrett, & Carter, 2013), we find that rangelands with higher NDVI values typically corresponded to more encroached vegetation states with low foraging value for cattle, while valuable open grasslands demonstrated consistently lower NDVI at any time of year than encroached vegetation states. In addition to revealing the phenological features of different vegetation types, the integrated approach has a potential to be widely adopted by local researchers where research infrastructure is relatively poor, because this approach relied completely on free data and analytical tools, and the entire research process is straightforward to replicate.

The application of state‐and‐transition model to hypothesize rangeland vegetation dynamics in Borana allows the identification of variability in the rates and pathways of encroachment among different vegetation states (Briske, 2017; D'Odorico et al., 2011), which differs from previous conceptions of homogenous encroachment (Angassa & Oba, 2008). Findings from our change detection analysis suggested that multiple transition pathways can occur simultaneously. However, there are several transition pathways that dominated the bush encroachment processes (Figure 5). At a higher elevation, the two dominant transition pathways are from open canopy woodland and bushland to dense scrubland. As the dominant class, dense scrubland demonstrated the greatest gain in area from 2003 to 2013. This indicates that ecological succession tends to stabilize upon reaching the dense scrubland state in the highland of Borana Zone, given relatively wet climatic conditions and the absence of fire (Mayer & Khalyani, 2011).

In the less arid highlands, large patches of open canopy woodland have been shifting into dense scrubland with dominant woody species such as *Acacia drepanolobium*,* A. nilotica* and *A. hockii*., because moderate to high grazing pressure removed understory herbaceous cover, while woody plant recruitment filled the open space between sparse trees. This represents a major transition pathway since fire was banned. The transition is expected to continue given the fact that nearly 35% open canopy woodland has disappeared in the past decade, mostly shifting to dense scrubland. In addition, the spatial distribution of open canopy woodland is getting patchier, often nested within dense scrubland, cultivated land and closed canopy woodland.

Another primary transition pathway is from bushland to dense scrubland, representing a shift from the second dominant class into the most dominant class. Although the average elevation of bushland is about 900 m, it has a wide altitudinal range, and those at the higher end of the range is shifting into dense scrubland. Given the ongoing climate change that is favorable to woody plant establishment (Kulmatiski & Beard, 2013), the plant recruitment process is facilitating the thickening of woody layer of bushland.

Although dense scrubland represents what is likely a very stable state, it could be converted into other classes under human‐imposed intervention (Gowda, Iams, & Silber, 2018). Dense scrublands, along with other minor classes such as closed and open canopy woodlands, that are situated at above 1,200 m are being converted to cultivated areas, which allows the practice of rain‐fed agriculture. Even until the 1950s, crop cultivation throughout the Borana Zone was banned by indigenous rules (Tache & Oba, 2010). Changes started in the 1980s, when a severe drought hit the zone. Pastoralists then began to fence rangelands and cultivate maize and teff to make ends meet. In recent years, commercial farming has become more prevalent, which has also contributed to cropland expansion at the expense of other rangeland vegetation classes.

One minor transition pathway in the highlands is from dense scrubland to closed canopy woodland. Given higher precipitation and absence of fire, tree seedlings in dense scrubland could grow into mature trees and gradually close the canopy (Mayer & Khalyani, 2011). Additionally, the government prohibited grazing in such forested areas for conservation purposes, which facilitated the transition into a closed vegetation state.

In the relatively dry lowlands of the Borana Zone, the primary vegetation transition is from grassland to bushland. Our change‐detection analysis revealed that over a quarter the grassland extent was lost over the 2003–2013 decade, making it the least extensive vegetation state among the eight identified. Once the dominant landscape in the lowlands (Cossins & Upton, 1987), it is being encroached by invasive species such as *A. mellifera* and *A. reficiens*, which could potentially change soil features and make it more difficult for grasses to grow (Rundel et al., 2014). Without fire, the encroaching woody species can continue to establish and eventually dominate the landscape, unless manual bush clearing intervention programs are implemented to thin the woody plant layer (Liao & Fei, 2017). The sparsely vegetated state occurs on lands where the unfavorable soils and other conditions severely limit vegetation presence and productivity. Consequently, transitions from this state to any of the others are highly improbable, as evidenced by its negligible change in extent over the 2003–2013 decade.

The identified transition pathways suggest potential opportunities for bush encroachment mitigation and rangeland management. In the highlands where the major pathways are from open canopy woodland and bushland to dense scrubland, a combination of browsing and fire can potentially reduce the woody plant layer and reverse the trend of encroachment (Staver, Bond, Stock, Rensburg, & Waldram, 2009). On the one hand, the graminoids and other herbs on the ground that are made inaccessible by dense shrubs and thickets for cattle grazing can serve as fuel loads to initiate a fire during the dry season. On the other hand, increasing browsing pressure by goats and camels can thin the woody plant layer and suppress the growth of shrubs and trees, which can indirectly facilitate the growth of herbs on the ground. In the drier lowlands where the transition pathway from grassland to bushland dominates, the chance to apply prescribed fire to control woody encroachment will be less feasible due to the lack of ground fuels (Archibald, Roy, Wilgen, & Scholes, 2009). In such locations, adding more goats and camels while reducing the number of cattle in the herds could be crucial to make better use of the woody forage resources that are well suited as food source for browsing (Liao, Ruelle, & Kassam, 2016). Rather than simply living with bush encroachment, pastoralists can actively contribute to its mitigation by changing their livestock portfolios.

## CONFLICT OF INTEREST

None declared.

## AUTHOR CONTRIBUTIONS

C.L., P.E.C, and S.D.D designed the study. C.L. conducted fieldwork, collected data, and performed data analysis. C.L., P.E.C, and S.D.D wrote the article.

## DATA ACCESSIBILITY

This publication is supported by MODIS NDVI data, which is publicly available at https://modis.gsfc.nasa.gov/data/dataprod/mod13.php. Field vegetation assessment data will be available for access on figshare.
