# Supplementary material for: Bush encroachment dynamics and rangeland management implications in southern Ethiopia
Source: Ecol Evol. 2018 Oct 26;8(23):11694–703. doi: 10.1002/ece3.4621 (PMC6303711; doi:10.1002/ece3.4621)
Supplement: Supplementary file 1 [file ECE3-8-11694-s001.docx]

**Supplementary Materials**

**Methods**

**Rangeland vegetation survey**: We conducted ground-truthing surveys in five study sites that represent a wide range of ecological zones in Borana. These five study sites are El Dima (latitude 4.3802; longitude: 37.2567; elevation: 841 m), Irbi (latitude: 4.0392; longitude: 38.6835; elevation: 1575 m), Shomo (latitude: 4.9366; longitude: 37.6196; elevation: 995 m), Siqu (latitude: 4.7615; longitude: 38.3916; elevation: 1486 m), and Taka Bulti (latitude: 4.5475; longitude: 39.0527; elevation: 1057 m). We conducted 27 vegetation survey at each study site and collected data on species composition and vegetation cover. We also collected a voucher specimen of each species and identified their scientific names at the National Herbarium of Ethiopia.

**Collection of geo-referenced photographs of rangeland vegetation**: We conducted field-based vegetation assessment to link remotely-sensed vegetation indices with contextualized vegetation classes in Borana. Specifically, we collected geo-referenced photos of representative rangeland vegetation classes throughout the entire Borana Zone, which can serve as the training and validation data in rangeland classification. A GPS-enabled camera was used to take photographs of rangelands in May-September, 2013, which spanned both wet and dry seasons. Due to logistic constraints, photos were acquired along roads and at specific sites. However, the sample we collected was distributed across the Borana Zone covering both low and high lands (Figure S1).

**Cross-validation with high-resolution satellite images**: Other than physiognomic features and species composition, the GPS-camera also recorded geographic coordinates and time. Thus, we generated point data from photo locations and used high-resolution imagery displays in Google Earth and Bing Map to examine and classify the 6.25-ha area surrounding each point. The vegetation types recorded by these photographs were classified based on the criteria in Table 1. We then examined the photos themselves to confirm or correct our initial classification.

**Inaccessible area assessment**: In addition to the photographs of field observations, we included 39 locations at the west and southwest corners of Borana Zone, which were inaccessible by a four-wheel drive vehicle. For these remote areas, we only used high-resolution Google Earth and Bing Map images to determine the vegetation classes. Our field-based evidence, coupled with satellite imagery, was intended to effectively address a spatial gap in vegetation mapping in one of the most remote regions of the world.

**Satellite imagery acquirement**: We used both spectral and temporal traits of satellite imageries to map the spatial distribution of existing vegetation states in the Borana Zone. Research in other dryland areas of Africa suggests the use of multiple compositing periods offers promise for broad-scale mapping of mixed woody and herbaceous vegetation (Higginbottom et al., 2018). In particular, multi-temporal datasets such as those from Moderate-resolution Imaging Spectroradiometer (MODIS) on board the Terra satellite, which is available for worldwide coverage since 2000, have made it possible to study vegetation dynamics in the most remote regions of the world. We used the processed Normalized Difference Vegetation Index (NDVI) images with a spatial resolution of 250 m and temporal resolution of 16 days acquired from MODIS, which has proven to be effective for rangeland classification (Piao et al., 2006). Since the training points were collected in the year of 2013, we used the 23 images from that year in our classification. We also obtained the 23 images in 2003 to derive the vegetation states a decade prior and compare those with 2013 conditions (Table S1).

**Phenology-based vegetation classification**: Vegetation phenology characterizes the periodic life-cycle events and their relation to climate, including the seasonal timing of vegetation growing seasons, canopy growth and senescence (Jones et al., 2011). While phenological events cannot be observed directly by satellite images, phenologically-related parameters such as the start, end, and length of the growing season can be estimated. NDVI metrics derived from temporal profile can separate different vegetation classes based on their phenology or seasonal signals (Linderholm, 2006).

In order to capitalize on phenological differences existing among differing vegetation states for rangeland classification, we conducted supervised classification using the non-parametric random forest classifier. The random forest algorithm is not constrained by parametric restrictions and is not sensitive to collinearity and overfitting of the data, allowing the use of layers with potential collinearity issues. The method takes random subsets from a training dataset and constructs classification trees using each of these subsets (Rodriguez-Galiano et al., 2012). The classification was performed using the randomForest package (Liaw & Wiener, 2002) in the R software environment (R Development Core Team, 2014).

In this study, the 23 NDVI images available throughout the year were considered 23 predictor variables. Rather than using a signal snapshot or an average of annual NDVI, these 23 images characterized the periodic life-cycle events of rangeland vegetation, including seasonal timing of growing seasons, canopy growth and senescence that can potentially distinguish each vegetation class. By using multiple observations of ‘greenness’ throughout the year, the phenological features of vegetation classes were taken into consideration in the supervised classification. We randomly selected 75% of ground-truth data for use in training the classifier, and the remainder were used to assess the accuracy of resultant classification map by constructing a classification error matrix.

**References**

Higginbottom, T. P., Symeonakis, E., Meyer, H., & van der Linden, S. (2018). Mapping fractional woody cover in semi-arid savannahs using multi-seasonal composites from Landsat data. *ISPRS Journal of Photogrammetry and Remote Sensing*, *139*, 88–102.

Jones, M. O., Jones, L. A., Kimball, J. S., & McDonald, K. C. (2011). Satellite passive microwave remote sensing for monitoring global land surface phenology. *Remote Sensing of Environment*, *115*(4), 1102–1114.

Liaw, A., & Wiener, M. (2002). Classification and regression by randomForest. *R News*, *2*(3), 18–22.

Linderholm, H. W. (2006). Growing season changes in the last century. *Agricultural and Forest Meteorology*, *137*(1–2), 1–14.

Piao, S., Mohammat, A., Fang, J., Cai, Q., & Feng, J. (2006). NDVI-based increase in growth of temperate grasslands and its responses to climate changes in China. *Global Environmental Change*, *16*(4), 340–348.

R Development Core Team. (2014). *R: A language and environment for statistical computing*. Vienna, Austria: R Foundation for Statistical Computing.

Rodriguez-Galiano, V. F., Ghimire, B., Rogan, J., Chica-Olmo, M., & Rigol-Sanchez, J. P. (2012). An assessment of the effectiveness of a random forest classifier for land-cover classification. *ISPRS Journal of Photogrammetry and Remote Sensing*, *67*, 93–104.

**Supplementary Tables**

Table S1. Forage plants and their palatability index to cattle, sheep, goats and camels.

| ID | Scientific Name | Family | Vernacular Name |
| --- | --- | --- | --- |
| 1 | *Barleria argentea* | *Acanthaceae* | *Agaggaroo harree* |
| 2 | *Barleria eranthemoides* | *Acanthaceae* | *Qilxiphee gaalaa* |
| 3 | *Barleria spinisepala* | *Acanthaceae* | *Qilxiphee gaalaa* |
| 4 | *Crabbea velutina* | *Acanthaceae* | *Qorsa gara/Baarata* |
| 5 | *Dicliptera verticillata* | *Acanthaceae* | *Unknown* |
| 6 | *Dyschoriste multicaulis* | *Acanthaceae* | *Gurbii* |
| 7 | *Hypoestes forskaolii* | *Acanthaceae* | *Darguu* |
| 8 | *Justicia cufodontii* | *Acanthaceae* | *Unknown* |
| 9 | *Justicia exigua* | *Acanthaceae* | *Gurbii daalaati* |
| 10 | *Justicia flava* | *Acanthaceae* | *Darguu* |
| 11 | *Justicia ornatopila* | *Acanthaceae* | *Saalima* |
| 13 | *Peristrophe paniculata* | *Acanthaceae* | *Darguu daalattii* |
| 13 | *Ruellia patula* | *Acanthaceae* | *Darguu adii* |
| 14 | *Aizoon canariense* | *Aizoaceae* | *Araddo* |
| 15 | *Zaleya pentandra* | *Aizoaceae* | *Araddo* |
| 16 | *Achyranthes aspera* | *Amaranthaceae* | *Darguu* |
| 17 | *Aerva javanica* | *Amaranthaceae* | *Habaaba* |
| 18 | *Alternanthera sessilis* | *Amaranthaceae* | *Mogorree* |
| 19 | *Amaranthus thunbergii* | *Amaranthaceae* | *Raafuu* |
| 30 | *Chenopodium opulifolium* | *Amaranthaceae* | *Ononnuu* |
| 31 | *Chionothrix latifolia* | *Amaranthaceae* | *Garbicha* |
| 33 | *Cyathula orhacantha* | *Amaranthaceae* | *Qulmuuduu* |
| 33 | *Nothosaerva brachiata* | *Amaranthaceae* | *Unknown* |
| 34 | *Psilotrichum gnaphalobryum* | *Amaranthaceae* | *Qorsa buutii* |
| 35 | *Pupalia lappacea* | *Amaranthaceae* | *Unknown* |
| 36 | *Sericocomopsis pallida* | *Amaranthaceae* | *Gurbii adii* |
| 37 | *Volkensinia prostrata* | *Amaranthaceae* | *Jilbeete* |
| 38 | *Crinum abyssinicum* | *Amaryllidaceae* | *Buttee waraabessaa* |
| 39 | *Lannea rivae* | *Anacardiaceae* | *Dakkidhaha/Handaraka* |
| 30 | *Ozoroa insignis* | *Anacardiaceae* | *Garrii* |
| 31 | *Rhus natalensis* | *Anacardiaceae* | *Daboobessa diidaa* |
| 33 | *Rhus tenuinervis* | *Anacardiaceae* | *Daboobessa* |
| 33 | *Peucedanum harmsianum* | *Apiaceae* | *Michuu* |
| 34 | *Acokanthera schimperi* | *Apocynaceae* | *Qaraaruu* |
| 35 | *Carissa spinarum* | *Apocynaceae* | *Makkaniisa* |
| 36 | *Gomphocarpus fruticosus* | *Apocynaceae* | *Unknown* |
| 37 | *Sarcostemma viminale* | *Apocynaceae* | *Hangayyaa* |
| 38 | *Leptadenia hastata* | *Asclepiadaceae* | *Kalaalaa* |
| 39 | *Pentarrhinum insipidum* | *Asclepiadaceae* | *Goorisa* |
| 40 | *Asparagus falcatus* | *Asparagaceae* | *Sariitii* |
| 41 | *Asparagus racemosus* | *Asparagaceae* | *Sariitii* |
| 43 | *Chlorophytum gallabatense* | *Asparagaceae* | *Miirtuu* |
| 43 | *Sansevieria erythraeae* | *Asparagaceae* | *Algee* |
| 44 | *Sansevieria ehrenbergii* | *Asparagaceae* | *Cakkee* |
| 45 | *Aspilia ciliata* | *Asteraceae* | *Hadaa* |
| 46 | *Aspilia mossambicensis* | *Asteraceae* | *Hadaa* |
| 47 | *Aspilia sp.* | *Asteraceae* | *Unknown* |
| 48 | *Athroisma gracile* | *Asteraceae* | *Unknown* |
| 49 | *Bidens biternata* | *Asteraceae* | *Coqooqitii* |
| 50 | *Bidens hildebrandtii* | *Asteraceae* | *Abunee* |
| 51 | *Blepharispermum fruticosum* | *Asteraceae* | *Baanyaa* |
| 53 | *Galinsoga parviflora* | *Asteraceae* | *Unknown* |
| 53 | *Guizotia zavattarii* | *Asteraceae* | *Unknown* |
| 54 | *Helichrysum forsskahlii* | *Asteraceae* | *Unknown* |
| 55 | *Helichrysum glumaceum* | *Asteraceae* | *Urri* |
| 56 | *Kleinia squarrosa* | *Asteraceae* | *Xixiixxuu* |
| 57 | *Lactuca inermis* | *Asteraceae* | *Baarata* |
| 58 | *Lactuca serriola* | *Asteraceae* | *Gurbii* |
| 59 | *Osteospermum monocephalum* | *Asteraceae* | *Hadaa* |
| 60 | *Pegolettia senegalensis* | *Asteraceae* | *Gurbii* |
| 61 | *Tagetes minuta* | *Asteraceae* | *Suunkii* |
| 63 | *Tarchonanthus camphortus* | *Asteraceae* | *Hadaaddoo* |
| 63 | *Vernonia cinerascens* | *Asteraceae* | *Qaxxee* |
| 64 | *Vernonia galamensis* | *Asteraceae* | *Gurbii onaa* |
| 65 | *Vernonia phillipsiae* | *Asteraceae* | *Darguu* |
| 66 | *Vernonia popeana* | *Asteraceae* | *Unknown* |
| 67 | *Cordia africana* | *Boraginaceae* | *Waddeessa* |
| 68 | *Cordia sinensis* | *Boraginaceae* | *Madheera raphachoo* |
| 69 | *Cynoglossum densefoliatum* | *Boraginaceae* | *Small hanqarre* |
| 70 | *Ehretia cymosa* | *Boraginaceae* | *Ulaagaa* |
| 71 | *Heliotropium zeylanicum* | *Boraginaceae* | *Awaayee diidaa* |
| 73 | *Boswellia neglecta* | *Burseraceae* | *Dakkara* |
| 73 | *Commiphora africana* | *Burseraceae* | *Hammeessa dhiiroo* |
| 74 | *Commiphora corrugata* | *Burseraceae* | *Siltaachoo* |
| 75 | *Commiphora erythraea* | *Burseraceae* | *Agarsuu* |
| 76 | *Commiphora habessinica* | *Burseraceae* | *Hoomachoo* |
| 77 | *Commiphora kua* | *Burseraceae* | *Callaanqaa* |
| 78 | *Commiphora schimperi* | *Burseraceae* | *Hammeessa dhiiroo* |
| 79 | *Commiphora terebinthina* | *Burseraceae* | *Sanga igguu/Xiraawaa* |
| 80 | *Bosica mossambicensis* | *Capparaceae* | *Qalqacha* |
| 81 | *Cadaba farinosa* | *Capparaceae* | *Mululacha* |
| 83 | *Cleome monophylla* | *Capparaceae* | *Shaanaa simphorree* |
| 83 | *Maerua sessiliflora* | *Capparaceae* | *Dhumasoo* |
| 84 | *Minuartia filifolia* | *Caryophyllaceae* | *Unknown* |
| 85 | *Pollichia campestris* | *Caryophyllaceae* | *Guunguma korbeessaa* |
| 86 | *Maytenus arbutifolia* | *Celastraceae* | *Mululacha* |
| 87 | *Maytenus parviflora* | *Celastraceae* | *Fonkolcha* |
| 88 | *Combretum hereroense* | *Combretaceae* | *Kennoo* |
| 89 | *Combretum molle* | *Combretaceae* | *Rukeessa* |
| 90 | *Terminalia brownii* | *Combretaceae* | *Rukeessa* |
| 91 | *Terminalia prunioides* | *Combretaceae* | *Qorobboo* |
| 93 | *Commelina africana* | *Commelinaceae* | *Qaayyoo* |
| 93 | *Commelina latifolia* | *Commelinaceae* | *Qaayyoo* |
| 94 | *Ipomoea donaldsonii* | *Convolvulaceae* | *Dhaliyyee* |
| 95 | *Ipomoea hildebrandtii* | *Convolvulaceae* | *Omborokkee* |
| 96 | *Ipomoea marmorata* | *Convolvulaceae* | *Obbee* |
| 97 | *Ipomoea obscura* | *Convolvulaceae* | *Dambiisuu* |
| 98 | *Ipomoea sp* | *Convolvulaceae* | *Maaracaa* |
| 99 | *Kalanchoe sp* | *Crassulaceae* | *Unknown* |
| 100 | *Cucumis dipsaceus* | *Cucurbitaceae* | *Baranbarroo* |
| 101 | *Momordica foetida* | *Cucurbitaceae* | *Buratee* |
| 103 | *Juniperus procera* | *Cupressaceae* | *Hidheessa* |
| 103 | *Cyperus amauropus* | *Cyperaceae* | *Quundhi* |
| 104 | *Cyperus dereilema* | *Cyperaceae* | *Illaadduu* |
| 105 | *Cyperus pulchellus* | *Cyperaceae* | *Daraaraa titee* |
| 106 | *Euclea divinorum* | *Ebenaceae* | *Mieessaa* |
| 107 | *Acalypha fruticosa* | *Euphorbiaceae* | *Dhirrii booranoo* |
| 108 | *Crodon dichogamus* | *Euphorbiaceae* | *Addaaddoo* |
| 109 | *Crodon macrostachyus* | *Euphorbiaceae* | *Makkaniisa* |
| 110 | *Euphorbia crotonoides* | *Euphorbiaceae* | *Uuroo* |
| 111 | *Euphorbia cuneata* | *Euphorbiaceae* | *Bursa* |
| 113 | *Euphorbia nubia* | *Euphorbiaceae* | *Aannoo woraabessaa* |
| 113 | *Euphorbia tirucalli* | *Euphorbiaceae* | *Aannoo loonii/surree* |
| 114 | *Ricinus communis* | *Euphorbiaceae* | *Qobboo* |
| 115 | *Tragia pungens* | *Euphorbiaceae* | *Dobbii* |
| 116 | *Acacia brevispica* | *Fabaceae* | *Hammareessa* |
| 117 | *Acacia bussei* | *Fabaceae* | *Hallo* |
| 118 | *Acacia drepanolobium* | *Fabaceae* | *Fuleensa* |
| 119 | *Acacia goetzei* | *Fabaceae* | *Burra* |
| 130 | *Acacia hockii* | *Fabaceae* | *Dabbasoo* |
| 131 | *Acacia horrida* | *Fabaceae* | *Riiga/Caacannee* |
| 133 | *Acacia mellifera* | *Fabaceae* | *Saphansa guraacha* |
| 133 | *Acacia nilotica* | *Fabaceae* | *Burquqqee* |
| 134 | *Acacia oerfota* | *Fabaceae* | *Waangaa* |
| 135 | *Acacia reficiens* | *Fabaceae* | *Sigirsoo* |
| 136 | *Acacia senegal* | *Fabaceae* | *Hidhaadhoo/Saphansa diimaa* |
| 137 | *Acacia seyal* | *Fabaceae* | *Waacu* |
| 138 | *Acacia sp* | *Fabaceae* | *Calloo* |
| 139 | *Acacia tortilis* | *Fabaceae* | *Dhaddacha* |
| 130 | *Calpurnia aurea* | *Fabaceae* | *Ceerataa* |
| 131 | *Crotalaria incana* | *Fabaceae* | *Unknown* |
| 133 | *Crotalaria pycnostachya* | *Fabaceae* | *Urri* |
| 133 | *Crotalaria ruspoliana* | *Fabaceae* | *Unknown* |
| 134 | *Dalbergia microphylla* | *Fabaceae* | *Wolchaamala* |
| 135 | *Delonix baccal* | *Fabaceae* | *Baalanjii* |
| 136 | *Delonix elata* | *Fabaceae* | *Sukeellaa* |
| 137 | *Dichrostachys cinerea* | *Fabaceae* | *Jirimee* |
| 138 | *Entada leptostachya* | *Fabaceae* | *Handaada* |
| 139 | *Indigofera schimperi* | *Fabaceae* | *Agaggaroo harree* |
| 140 | *Indigofera spicata* | *Fabaceae* | *Agaggaroo harree/Urra* |
| 141 | *Indigofera vohemarensis* | *Fabaceae* | *Agaggaroo harree* |
| 143 | *Indigofera volkensii* | *Fabaceae* | *Gurbii hoolaa* |
| 143 | *Ormocarpum trichocarpum* | *Fabaceae* | *Buutiyyee* |
| 144 | *Senna didymobotrya* | *Fabaceae* | *Unknown* |
| 145 | *Zornia apiculata* | *Fabaceae* | *Unknown* |
| 146 | *Albuca abyssinica* | *Hyacinthaceae* | *Unknown* |
| 147 | *Kirkia burgeri* | *Kirkiaceae* | *Bisdhugaa* |
| 148 | *Isodon ramosissimus* | *Lamiaceae* | *Qorsa buutii* |
| 149 | *Leucas cuneifolia* | *Lamiaceae* | *Darguu* |
| 150 | *Leucas stachydiformis* | *Lamiaceae* | *Unknown* |
| 151 | *Ocimum americanum* | *Lamiaceae* | *Dama kasee* |
| 153 | *Ocimum forskolei* | *Lamiaceae* | *Hancabbii* |
| 153 | *Ocimum stirbeyi* | *Lamiaceae* | *Urgoo dhadhaa* |
| 154 | *Ocimum urticifolium* | *Lamiaceae* | *Hancabbii* |
| 155 | *Plectranthus caninus* | *Lamiaceae* | *Harcaaa* |
| 156 | *Plectranthus igniarius* | *Lamiaceae* | *Barbaaressa* |
| 157 | *Plectranthus tenuiflorus* | *Lamiaceae* | *Barbaaressa* |
| 158 | *Premna schimperi* | *Lamiaceae* | *Xaaxessaa* |
| 159 | *Erianthemum aethiopicum* | *Loranthaceae* | *Dirreetto* |
| 160 | *Emelianthe panganensis* | *Loranthaceae* | *Hidha* |
| 161 | *Abutilon figarianum* | *Malvaceae* | *Babaaliyyoo* |
| 163 | *Abutilon fruticosum* | *Malvaceae* | *Babaaliyyoo* |
| 163 | *Abutilon graveolens* | *Malvaceae* | *Gurbii baddaa* |
| 164 | *Grewia bicolor* | *Malvaceae* | *Harooressa* |
| 165 | *Grewia lilacina* | *Malvaceae* | *Harooressa* |
| 166 | *Grewia pennicillata* | *Malvaceae* | *Harooressa bosoqee* |
| 167 | *Grewia tembensis* | *Malvaceae* | *Dheekkaa* |
| 168 | *Grewia velutina* | *Malvaceae* | *Harooressa hiddoo* |
| 169 | *Grewia villosa* | *Malvaceae* | *Ogomdii* |
| 170 | *Hibiscus boranensis* | *Malvaceae* | *Bungaala* |
| 171 | *Hibiscus crassinervius* | *Malvaceae* | *Bungaala* |
| 173 | *Hibiscus ovalifolius* | *Malvaceae* | *Gurbii daalatii* |
| 173 | *Hibiscus sparseaculeatus* | *Malvaceae* | *Dunuunnuu* |
| 174 | *Melhania sp* | *Malvaceae* | *Gaadii beeraa* |
| 175 | *Pavonia erlangeri* | *Malvaceae* | *Iccinnii/Gurbii diimtuu* |
| 176 | *Sida ovata* | *Malvaceae* | *Bungaala adii* |
| 177 | *Sterculia stencarpa* | *Malvaceae* | *Qararrii* |
| 178 | *Triumfetta flavescens* | *Malvaceae* | *unknown* |
| 179 | *Triumfetta heterocarpa* | *Malvaceae* | *Hanqarree* |
| 180 | *Ficus sycomorus* | *Moraceae* | *Qilxaa* |
| 181 | *Ficus vasta* | *Moraceae* | *Rabbana* |
| 183 | *Commicarpus plumbagineus* | *Nyctaginaceae* | *Unknown* |
| 183 | *Jasminum fluminense* | *Oleaceae* | *Aadee baddaa* |
| 184 | *Olea europaea* | *Oleaceae* | *Ejersa* |
| 185 | *Sesamothamnus rivae* | *Pedaliaceae* | *Lalaaftoo* |
| 186 | *Aristida adoensis* | *Poaceae* | *Marra saalaa/Saattuu biilaa* |
| 187 | *Aristida kenyensis* | *Poaceae* | *Biilaa diidaa* |
| 188 | *Aristida mutabilis* | *Poaceae* | *Biilaa gaaree* |
| 189 | *Bothriochloa insculpta* | *Poaceae* | *Unknown* |
| 190 | *Cenchrus ciliaris* | *Poaceae* | *Mata guddesessa* |
| 191 | *Chloris pycnothrix* | *Poaceae* | *Unknown* |
| 193 | *Chloris roxburghiana* | *Poaceae* | *Buuyoo gooloo* |
| 193 | *Chloris virgata* | *Poaceae* | *Unknown* |
| 194 | *Chrysopogon auheri* | *Poaceae* | *Alaloo* |
| 195 | *Cynodon plectostachyus* | *Poaceae* | *Ardaa* |
| 196 | *Digitaria pennata* | *Poaceae* | *Maansilee* |
| 197 | *Eleusine intermedia* | *Poaceae* | *Coqorsa* |
| 198 | *Enneapogon persicus* | *Poaceae* | *Alaloo/Imogori* |
| 199 | *Enteropogon macrostachyus* | *Poaceae* | *Biilaa* |
| 200 | *Eragrostis capitulifera* | *Poaceae* | *Marra bukicha* |
| 201 | *Eragrostis cilianensis* | *Poaceae* | *Bukicha* |
| 202 | *Eragrostis cylindriflora* | *Poaceae* | *Unknown* |
| 203 | *Eragrostis minor* | *Poaceae* | *Unknown* |
| 204 | *Eragrostis papposa* | *Poaceae* | *Unknown* |
| 205 | *Eragrostis sennii* | *Poaceae* | *Unknown* |
| 206 | *Eustachys paspaloides* | *Poaceae* | *Coqorsa* |
| 207 | *Harpachne schimperi* | *Poaceae* | *Billaa* |
| 208 | *Heteropogon contortus* | *Poaceae* | *Seericha* |
| 209 | *Hyparrhenia anamesa* | *Poaceae* | *Buyyoo gaaraa* |
| 210 | *Leptochloa obtusiflora* | *Poaceae* | *Laabbessa* |
| 211 | *Melinis repens* | *Poaceae* | *Halciisoo* |
| 212 | *Panicum maximum* | *Poaceae* | *Loloqaa* |
| 213 | *Panicum repens* | *Poaceae* | *Loloqaa* |
| 214 | *Pennisetum glaucifolium* | *Poaceae* | *Ogoondhicho* |
| 215 | *Pennisetum mezianum* | *Poaceae* | *Ogoondhicho* |
| 216 | *Pennisetum thunbergii* | *Poaceae* | *Gursumesa* |
| 217 | *Setaria incrassata* | *Poaceae* | *Ogoondhicho* |
| 218 | *Setaria verticillata* | *Poaceae* | *Raaphuphaa* |
| 219 | *Sporobolus festivus* | *Poaceae* | *Salaqoo* |
| 220 | *Sporobolus nervosus* | *Poaceae* | *Salaqoo* |
| 221 | *Sporobolus pyramidalis* | *Poaceae* | *Luucolee* |
| 222 | *Sporobolus spicatus* | *Poaceae* | *Hiddoo diidaa* |
| 223 | *Tetrapogon cenchriformis* | *Poaceae* | *Salaqoo/bukicha* |
| 224 | *Themeda triandra* | *Poaceae* | *Gaaguroo* |
| 225 | *Tragus berteronianus* | *Poaceae* | *Marra samphalle* |
| 226 | *Oxygonum sinuatum* | *Polygonaceae* | *Mogorre* |
| 227 | *Caylusea abyssinica* | *Resedaceae* | *Darguu* |
| 228 | *Psydrax schimperiana* | *Rubiaceae* | *Gaallee* |
| 229 | *Dobera glabra* | *Salvadoraceae* | *Garsee* |
| 230 | *Salvadora persica* | *Salvadoraceae* | *Aadee* |
| 231 | *Osyris quadripartita* | *Santalaceae* | *Waatoo* |
| 232 | *Dodonaea angustifolia* | *Sapindaceae* | *Dhitacha* |
| 233 | *Haplocoelum foliolosum* | *Sapindaceae* | *Canaa* |
| 234 | *Pappea capensis* | *Sapindaceae* | *Biiqqaa* |
| 235 | *Cycnium herzfeldianum* | *Scrophulariaceae* | *Unknown* |
| 236 | *Datura stramonium* | *Solanaceae* | *Qobboo* |
| 237 | *Discopodium penninervium* | *Solanaceae* | *Hiddii xirooftuu* |
| 238 | *Solanum giganteum* | *Solanaceae* | *Hiddii loonii* |
| 239 | *Solanum marginatum* | *Solanaceae* | *Hiddii fardaa* |
| 240 | *Solanum schimperianum* | *Solanaceae* | *Hiddii qixii/ re’ee* |
| 241 | *Solanum somalense* | *Solanaceae* | *Hiddii gaagee* |
| 242 | *Withania somnifera* | *Solanaceae* | *Hiddii* |
| 243 | *Gnidia somalensis* | *Thymelaceae* | *Midhiddi didiqqo* |
| 244 | *Xerophyta humilis* | *Velloziaceae* | *Areedoo* |
| 245 | *Lantana camara* | *Verbenaceae* | *Midhaan dubraa* |
| 246 | *Lippia carviodora* | *Verbenaceae* | *Urgoo loonii* |
| 247 | *Cissus quadrangularis* | *Vitaceae* | *Cophii soodduu* |
| 248 | *Cissus rotundifolia* | *Vitaceae* | *Cophii kooraa* |
| 249 | *Cyphostemma boranense* | *Vitaceae* | *Cophii arbaa* |
| 250 | *Cyphostemma ternatum* | *Vitaceae* | *Cophii tutufi* |
| 251 | *Aloe pirottae* | *Xanthorrhoeaceae* | *Hargeessa dhalaa* |
| 252 | *Balanites aegyptiaca* | *Zygophyllaceae* | *Baddana luoo* |

Table S2. Date of NDVI images in 2003 and 2013 used in rangeland vegetation classification.

| Image ID | Date |
| --- | --- |
| 1 | 01/01 |
| 2 | 01/17 |
| 3 | 02/02 |
| 4 | 02/18 |
| 5 | 03/06 |
| 6 | 03/22 |
| 7 | 04/07 |
| 8 | 04/23 |
| 9 | 05/09 |
| 10 | 05/25 |
| 11 | 06/10 |
| 12 | 06/26 |
| 13 | 07/12 |
| 14 | 07/28 |
| 15 | 08/13 |
| 16 | 08/29 |
| 17 | 09/14 |
| 18 | 09/30 |
| 19 | 10/16 |
| 20 | 11/01 |
| 21 | 11/17 |
| 22 | 12/03 |
| 23 | 12/19 |

Table S3. The confusion matrix of classification results.

| Vegetation class | CCW | DS | BU | OCW | SS | CL | GR | SV | User’s Accuracy |
| --- | --- | --- | --- | --- | --- | --- | --- | --- | --- |
| CCW | 20 | 0 | 0 | 0 | 0 | 0 | 0 | 0 | 100.0% |
| DS | 0 | 133 | 8 | 22 | 8 | 5 | 0 | 0 | 75.6% |
| BU | 0 | 6 | 82 | 6 | 3 | 0 | 0 | 0 | 84.5% |
| OCW | 0 | 7 | 2 | 37 | 9 | 4 | 0 | 0 | 62.7% |
| SS | 0 | 6 | 2 | 5 | 39 | 4 | 0 | 0 | 69.6% |
| CL | 1 | 4 | 1 | 2 | 3 | 23 | 0 | 0 | 67.6% |
| GR | 0 | 0 | 1 | 0 | 1 | 0 | 16 | 0 | 88.9% |
| SV | 0 | 0 | 0 | 0 | 0 | 0 | 0 | 1 | 100.0% |
| Producer’s Accuracy | 95.2% | 85.3% | 85.4% | 51.4% | 61.9% | 63.9% | 100.0% | 100.0% | 76.1% |

Footnote: Among the eight vegetation classes, grassland and sparsely vegetated land were perfectly validated according to producer’s accuracy. Closed canopy woodland had an accuracy over 95%. The two dominant vegetation classes, namely dense scrubland and bushland, were also well classified, with an accuracy index over 85%. The remaining three classes were less accurately classified. Among the 36 cultivated land validation points, 13 were misclassified as open canopy woodland, dense or sparse scrubland. This was likely because cultivated land was usually converted from these three vegetation classes, thus often nested within them. In addition, since the areal extent of cultivated land was usually less than the NDVI cell size (6.25 hectares) in many cases, they were more difficult to distinguish at that resolution. Sparse scrubland showed a similar accuracy index, probably because it was often confused with open canopy woodland at the lower end of woody plant cover and dense scrubland at the higher end. The lowest accuracy was observed with open canopy woodland, which was the typical savanna-like type. It was most likely to be confused with dense scrubland, into which this vegetation class seemed to be shifting.

Table S4. Vegetation transition matrix in the 2003-2013 decade (unit = km^2^).

| Vegetation Class | CCW | DS | BU | OCW | SS | CL | GR | SV | 2003 Total |
| --- | --- | --- | --- | --- | --- | --- | --- | --- | --- |
| CCW | 2318 | 823 | 35 | 1 | 5 | 39 | 0 | 0 | 3221 |
| DS | 935 | 19093 | 1690 | 578 | 233 | 871 | 33 | 0 | 23433 |
| BU | 29 | 3077 | 7367 | 121 | 221 | 28 | 158 | 1 | 11003 |
| OCW | 10 | 1191 | 248 | 629 | 236 | 185 | 16 | 0 | 2514 |
| SS | 1 | 709 | 379 | 189 | 237 | 72 | 27 | 0 | 1613 |
| CL | 2 | 355 | 18 | 124 | 19 | 129 | 1 | 0 | 647 |
| GR | 0 | 0 | 398 | 6 | 7 | 1 | 218 | 7 | 637 |
| SV | 0 | 0 | 0 | 0 | 0 | 0 | 11 | 622 | 634 |
| 2013 Total | 3295 | 25248 | 10134 | 1648 | 957 | 1325 | 464 | 631 | 43703 |

**Supplementary Figures**


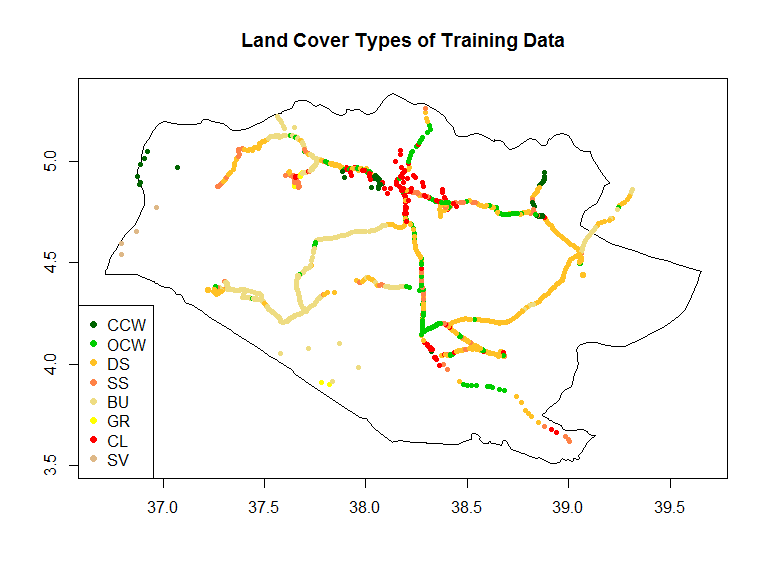


Figure S1. Locations of field data collection and vegetation classes.


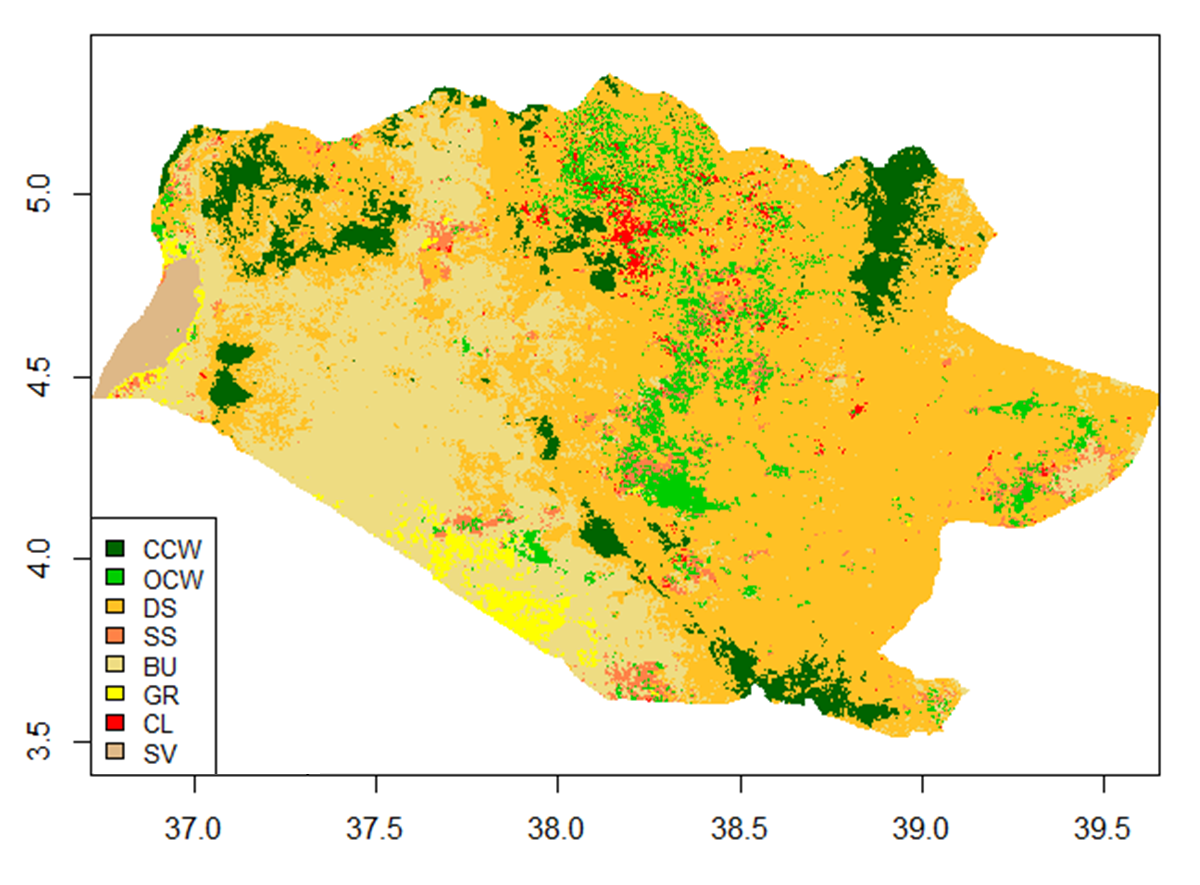


Figure S2. Vegetation classes and their spatial distribution in 2003.
